# Fusing Positive and Negative CT Contrast Nanoagent for the Sensitive Detection of Hepatoma

**DOI:** 10.1002/advs.202304668

**Published:** 2023-10-23

**Authors:** Xianfu Meng, Jiahao Gao, Yanhong Sun, Fei Duan, Bixue Chen, Guanglei Lv, Huiyan Li, Xingwu Jiang, Yelin Wu, Jiawen Zhang, Xiangming Fang, Zhenwei Yao, Changjing Zuo, Wenbo Bu

**Affiliations:** ^1^ Department of Nuclear Medicine Changhai Hospital Navy Medical University Shanghai 200433 China; ^2^ Department of Materials Science and State Key Laboratory of Molecular Engineering of Polymers Academy for Engineering and Technology Fudan University Shanghai 200433 China; ^3^ Department of Radiology Huashan Hospital Fudan University Shanghai 200040 China; ^4^ Department of Gastroenterology Changhai Hospital Naval Medical University Shanghai 200433 China; ^5^ Department of Radiology Eye & ENT Hospital of Fudan University Fudan University Shanghai 200031 China; ^6^ Department of Radiology Wuxi People's Hospital Nanjing Medical University Wuxi 214023 China; ^7^ Center for Biotechnology and Biomedical Engineering Yiwu Research Institute of Fudan University Yiwu 322000 China; ^8^ Tongji University Cancer Center Shanghai Tenth People's Hospital Tongji University School of Medicine Shanghai 200072 China

**Keywords:** hepatoma, Hf‐MOF, negative CT contrast, positive CT contrast

## Abstract

Positive computed tomography (CT) contrast nanoagent has significant applications in diagnosing tumors. However, the sensitive differentiation between hepatoma and normal liver tissue remains challenging. This challenge arises primarily because both normal liver and hepatoma tissues capture the nanoagent, resulting in similar positive CT contrasts. Here, a strategy for fusing positive and negative CT contrast nanoagent is proposed to detect hepatoma. A nanoagent Hf‐MOF@AB@PVP initially generates a positive CT contrast signal of 120.3 HU in the liver. Subsequently, it can specifically respond to the acidic microenvironment of hepatoma to generate H_2_, further achieving a negative contrast of −96.0 HU. More importantly, the relative position between the negative and positive signals area is helpful to determine the location of hepatoma and normal liver tissues. The distinct contrast difference of 216.3 HU and relative orientation between normal liver and tumor tissues are meaningful to sensitively distinguish hepatoma from normal liver tissue utilizing CT imaging.

## Graphical abstract

A strategy for fusing positive and negative CT contrast nanoagent is presented. The distinct contrast of 216.3 HU and relative orientation between positive and negative CT signals are meaningful to sensitively distinguish hepatoma from normal liver tissue utilizing CT.

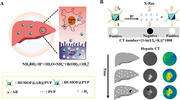

## 1 Introduction

During CT scanning, most contrast agents commonly used in clinics including iodine agents produce high CT values, which are generally defined as positive CT contrast agents.^[^ ^1^ ^]^ Among these, positive CT contrast nanoagents have many merits in detecting tumors and the microenvironment, such as versatile designability and enhanced permeability and retention (EPR) effect.^[^ ^2^ ^]^ However, positive CT contrast nanoagents still face significant challenges in sensitively detecting liver cancer. One reason is that tumor heterogeneity leads to the non‐specific distribution of CT contrast nanoagents in the liver.^[^ ^3^ ^]^ The nanoagents are captured by both hepatoma tissues and normal liver tissues, including plenty of Kupffer cells.^[^ ^4^ ^]^ As a result, similar enhanced CT contrast signals are observed in hepatoma tissues and normal liver tissues, making it difficult to sensitively distinguish between them.

The lunar eclipse is a special astronomical phenomenon. When the moon travels to the shaded part of the Earth, it displays a unique contrast between brightness and darkness.^[^ ^5^ ^]^ Inspired by this, if the “darkness” can specifically appear near the hepatoma tissues with “brightness” signals, the hepatoma tissues will be distinguished. In 2019, our group proposed the concept of negative CT contrast nanoagents,^[^ ^6^ ^]^ making it possible to detect solid tumors without cavities by using gas as CT contrast agent. Negative CT contrast agents are a kind of substances that have low density, weak attenuation of X‐ray, and show negative contrast signals on CT images. Therefore, if a nanoagent can exhibit both positive and negative CT contrast properties, resembling a “lunar eclipse” within the hepatoma, it will enable the sensitive detection of hepatoma tissues.

**Scheme 1:**
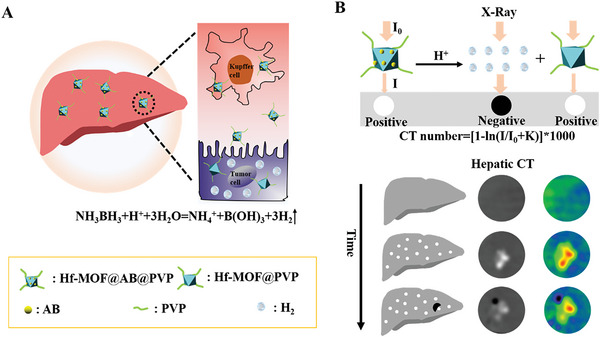
Fusing positive and negative CT contrast nanoagents for the sensitive detection of hepatoma.

Here, a strategy was proposed to sensitively diagnose the hepatoma by fusing positive and negative CT contrast nanoagent. The designed nanoagent was capable of generating both positive and negative CT contrast signals simultaneously at the hepatoma. It is more significant that the relative position between the positive and negative CT contrast signals contributed to determining the location of hepatoma and normal tissues (**Scheme** **1**). First, a nanoagent Hf‐MOF@AB@PVP was prepared, which contained a hafnium‐based metal–organic framework (MOF) with a high atomic number, aminoborane (AB) for generating H_2_ in response to acidity, and polyvinylpyrrolidone (PVP) to enhance the biocompatibility. When the nanoagent was administered into the body, it mainly accumulated in the liver. It initially presented a positive CT contrast signal of 120.3 HU because of the element Hf with a high atomic number (72). However, both tumor and normal liver tissues swallowed the contrast nanoagent and exhibited high CT contrast, which made it challenging to delineate the tumor's location. Over time, the contrast nanoagent in the tumor site specifically responded to the acidic microenvironment, releasing H_2_ and conducting a CT signal value of −96.0 HU. The signal difference between the negative signal area and the positive signal area was 216.3 HU, forming a distinct contrast. More importantly, the relative position between the negative and positive signals area was helpful in determining the location of the tumor and normal tissues. This makes the nanoagent an ideal candidate for the sensitive detection of soft tissue lesions by CT imaging.

## 2 Results

### 2.1 Synthesis and Characterization of Hf‐MOF@AB@PVP Nanoparticles

**Figure 1:**
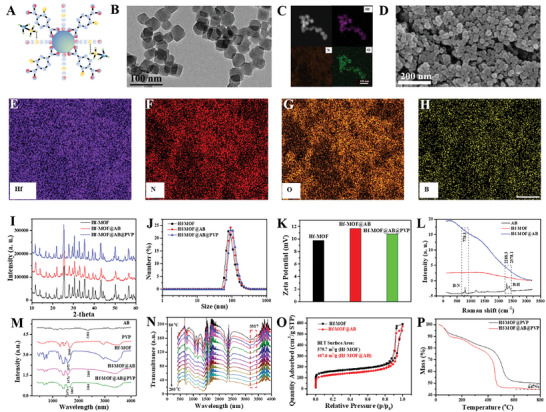
Characterization of Hf‐MOF@AB@PVP nanoparticles. A) A diagram of Hf‐MOF@AB@PVP nanoparticles; B,C) TEM and element mapping images of Hf‐MOF@AB@PVP nanoparticles, scale bar was 100 nm; D) SEM image of Hf‐MOF@AB@PVP nanoparticles, scale bar was 200 nm; E–H) Element mapping of Hf, N, O, B in SEM images, scale bar was 2.5 µm; I) XRD spectra of Hf‐MOF, Hf‐MOF@AB and Hf‐MOF@AB@PVP nanoparticles; J,K) DLS and Zeta‐potential results of Hf‐MOF, Hf‐MOF@AB and Hf‐MOF@AB@PVP nanoparticles; L) Raman spectra of AB, Hf‐MOF and Hf‐MOF@AB; M) FT‐IR spectra of AB, PVP, Hf‐MOF, Hf‐MOF@AB and Hf‐MOF@AB@PVP nanoparticles; N) In situ FT‐IR spectra with a varied temperature of Hf‐MOF@AB@PVP nanoparticles; O) Nitrogen adsorption and desorption results of Hf‐MOF and Hf‐MOF@AB nanoparticles; P) Thermogravimetry (TG) tests of Hf‐MOF@AB and Hf‐MOF@AB@PVP nanoparticles.

Hf‐MOF@AB@PVP nanoparticles were successfully prepared according to a modified method (Figure S1, Supporting Information).^[^ ^7^ ^]^ Hf‐MOF nanoparticles had a uniform diameter, as observed in the transmission electron microscope (TEM) (Figure S2, Supporting Information) and scanning electron microscope (SEM) (Figure S3, Supporting Information) images. AB molecules were then loaded into the pores of Hf‐MOF nanoparticles by hydrogen bond interaction. The morphology and size of the nanoparticles changed little from the TEM (Figure S4, Supporting Information) and SEM (Figure S5, Supporting Information) images. Next, PVP molecules were decorated onto the surface of Hf‐MOF@AB nanoparticles, resulting in the formation of Hf‐MOF@AB@PVP nanoparticles (**Figure** **1**A). As shown in Figure 1B, TEM images showed that Hf‐MOF@AB@PVP nanoparticles had an approximate diameter of 45.2 nm. Element mapping by TEM exhibited the presence of Hf, N, and O elements on the nanoparticles (Figure 1C). However, due to the resolution limitations of TEM, the B element was not shown. Therefore, SEM images were employed, showing a monodispersed and uniform morphology of Hf‐MOF@AB@PVP nanoparticles (Figure 1D). Element mapping images showed all of the B, N, O, and Hf elements on the nanoparticles, which demonstrated the successful synthesis (Figure 1E–H). X‐ray photoelectron spectroscopy (XPS) spectra also demonstrated the existence of B, C, N, O, and Hf elements (Figure S6, Supporting Information). X‐ray diffraction (XRD) results further proved the successful synthesis and minimal impact on the crystallinity after PVP modification (Figure 1I). Dynamic light scattering (DLS) results indicated a hydrodynamic diameter of 91.3 nm (Figure 1J), showing little difference among the three groups. Zeta potential exhibited an increase after loading AB molecules and a subsequent decrease after PVP modification (Figure 1K).

Subsequently, both Raman spectra (Figure 1L) and Fourier transform infrared (FT‐IR) spectra (Figure 1M) demonstrated the successful loading of AB molecules and the modification of PVP molecules. In addition, in situ FT‐IR spectra demonstrated the gradual disappearance of the wavelength at 3317 nm with increasing temperature (Figure 1N), clarifying the hydrogen bonding function between Hf‐MOF and AB molecules. Furthermore, the results of nitrogen adsorption and desorption experiments displayed a decrease of approximately 19.3% in the specific surface area of Hf‐MOF after loading AB molecules (Figure 1O). Meanwhile, the t‐plot micropore areas decreased by about 31.2%, while the t‐plot external surface areas changed few (Figure S7, Supporting Information). It indicated that AB molecules were successfully loaded into the pores. Finally, thermogravimetry analysis (TGA) demonstrated that approximately 4.67% (in mass) of AB molecules were loaded in the pores of Hf‐MOF nanoparticles (Figure 1P).

### 2.2 H_2_ Release and CT Imaging of Hf‐MOF@AB@PVP Nanoparticles in Aqueous Solutions

**Figure 2:**
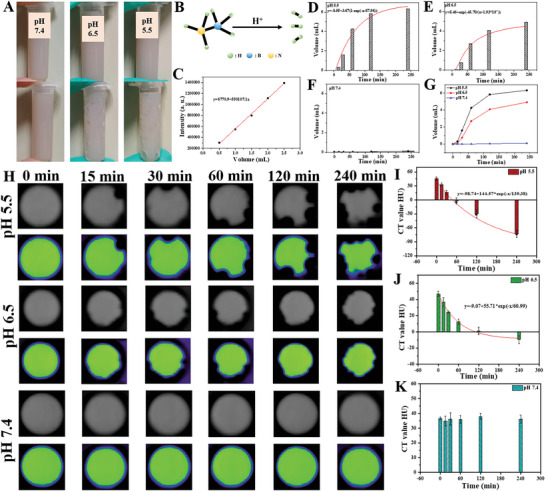
A) Photos of Hf‐MOF@AB@PVP nanoparticles in different pH values; B) A chart of ammonia borane reacting with an acid to generate H_2_; C) A standard curve of H_2_; D–F) Histograms of H_2_ generation and dynamic simulation of Hf‐MOF@AB@PVP nanoparticles in different pH values; G) Line chat of H_2_ generation; H) Grayscale and pseudo‐color CT images of Hf‐MOF@AB@PVP nanoparticles in different pH values and different time; I–K) Histograms of CT value in different pH values and different time.

First, the photographs of the H_2_ release of Hf‐MOF@AB@PVP nanoparticles in different pH values (pH = 7.4, 6.5, and 5.5) were acquired (**Figure** **2**A). A large amount of H_2_ was released in the acidic solutions, with higher acidity leading to a greater H_2_ generation. In contrast, minimal H_2_ generation was observed in the solution under pH 7.4. A schematic diagram illustrating the reaction of ammonia borane with acid to generate H_2_ was presented in Figure 2B. Subsequently, a gas chromatography system was employed to assess the H_2_ generation capability of Hf‐MOF@AB@PVP nanoparticles. A standard curve of H_2_ was first plotted (Figure 2C). Similar to the photographs of H_2_ release, gas chromatography showed that H_2_ was produced fast and slightly fast in the solution with pH 5.5 (Figure 2D) and pH 6.5 (Figure 2E). However, negligible H_2_ was detected in the solution with pH 7.4 (Figure 2F). 4 hours later, the AB molecules were almost decomposed, and the H_2_ release amount hardly increased at pH 5.5 and pH 6.5. After fitting, the release curves were suitable for the exponential growth model (Figure 2D,E). At first, a large number of H^+^ were enriched in the micro/mesoporous channels. The accelerated reaction rate between H^+^ and AB molecules might be attributed to the confinement effect, resulting in the rapid decomposition of AB to release H_2_. As the AB molecules approached complete decomposition, the reaction rate gradually decreased. The line chart reaffirmed that H_2_ generation was the fastest in the solution with pH 5.5, followed by the solution with pH 6.5. While minimal H_2_ generation occurred in the solution under pH 7.4 (Figure 2G).

The CT imaging capability of Hf‐MOF@AB@PVP nanoparticles and clinical iodine at different concentrations is shown in Figure S8 (Supporting Information). Grayscale images revealed a noticeable enhancement in CT contrast performance with increasing concentration from 125 to 2000 ppm. Pseudo‐color images showed a clearer contrast. The histograms of the corresponding CT value of Hf‐MOF@AB@PVP nanoparticles and clinical iodine (iopromide) at different concentrations were exhibited in Figures S9 and S10 (Supporting Information). For Hf‐MOF@AB@PVP, the CT value ranged from 10.1 to 152.9 HU as the concentration increased from 125 to 2000 ppm. For Hf‐MOF@AB@PVP, the CT value ranged from 15.0 to 73.3 HU as the concentration increased from 125 to 2000 ppm. Meanwhile, the dot plot indicated that the CT contrast performance of Hf‐MOF@AB@PVP nanoparticles and clinical iodine was 76.6 HU/(g/L) and 31.8 HU/(g/L) (Figure S11, Supporting Information), respectively.

Subsequently, the CT imaging contrast capability of Hf‐MOF@AB@PVP nanoparticles (500 ppm) was investigated at different pH (pH 5.5, 6.5, and 7.4) (Figure 2H). First, the nanoparticles exhibited an obvious positive CT imaging contrast capability. Since H_2_ has an extremely low density, it possesses a significantly negative CT contrast ability. Consequently, Hf‐MOF@AB@PVP nanoparticles demonstrated both positive and negative CT contrast performance in acidic solutions simultaneously, enormously improving CT imaging contrast. Over time, the negative CT contrast turned out to be more significant and the CT values decreased greatly under pH 5.5 and 6.5. The positive CT contrast images were gradually missing under pH 5.5 and 6.5, while they remained relatively stable under pH 7.4. This demonstrated the nanoparticles’ specific response to acid. Simultaneously, the corresponding histograms exhibited the specific CT values of Hf‐MOF@AB@PVP nanoparticles under different pH values. Under pH 5.5, the CT value ranged from 45.8 to −72.9 HU with a change of 118.7 HU (Figure 2I). Under pH 6.5, the CT value ranged from 46.9 to −9.6 HU with a change of 56.5 HU (Figure 2J). However, the CT value remained nearly unchanged under pH 7.4 (Figure 2K). Furthermore, it was found that the CT value in pH 5.5 and pH 6.5 showed exponential attenuation, which was similar to the trend of H_2_ generation. Based on the changing trend of CT value, it was possible to predict the pH value of the acid solution.

### 2.3 CT Imaging of Hf‐MOF@AB@PVP Nanoparticles in Subcutaneous Xenograft Tumor Models

**Figure 3:**
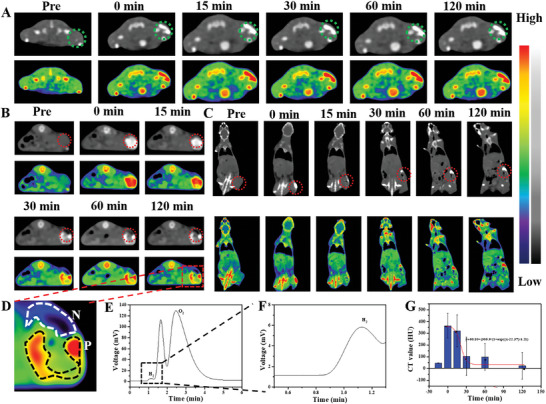
Transverse section CT images of mice injected with Hf‐MOF@AB@PVP nanoparticles in muscle (*n* = 3) at different times (A), the green circle was the region of interest; Transverse (B) and coronal (C) section CT images of mice (*n* = 3) intratumorally injected with Hf‐MOF@AB@PVP nanoparticles in a subcutaneous xenograft tumor model and in a different time, the red circle was the region of interest; An amplified CT image of the tumor at 120 min (D), the white circle was negative signals area (N: negative) and the black circle was positive signals area (P: positive); E) A gas chromatography line of dissected tumor tissue; F) Enlarged image of the line in the region of interest; G) Histograms of CT value in the region of interest and different time.

The CT imaging capability of Hf‐MOF@AB@PVP nanoparticles was subsequently investigated in vivo. First, the nanoparticles were injected into the muscle of mice (**Figure** **3**A). Notably, immediate enhancement of CT contrast was observed in the region of interest (green circle) following the injection (transverse section). Besides, grayscale and pseudo‐color images showed that the CT imaging contrast could be still maintained over time without the generation of gas. This phenomenon further demonstrated that the nanoparticles would not be decomposed to produce H_2_ in normal tissues. At the same time, the coronal section CT images also showed that Hf‐MOF@AB@PVP nanoparticles had a significantly positive CT contrast ability, while they had no ability to generate H_2_ in muscle (Figure S12, Supporting Information).

Next, Hf‐MOF@AB@PVP nanoparticles were injected into the tumor. It was observed that the tumor tissue in the region of interest (red circle) was lighted immediately in the grayscale images (transverse section) (Figure 3B). Pseudo‐color images more clearly showed the enhanced contrast. Over time (0‐120 min), a dark area gradually appeared and expanded within the tumor tissue. This phenomenon was attributed to the release of H_2_ from Hf‐MOF@AB@PVP nanoparticles in the acidic tumor tissue. Similarly, the coronal section CT images in the region of interest (red circle) not only demonstrated a significantly positive CT contrast ability of Hf‐MOF@AB@PVP nanoparticles but also had the ability to generate H_2_ in response to the acidic tumor microenvironment (Figure 3C). More importantly, as shown in the enlarged image (Figure 3D), the remarkable contrast and the relative position between the positive and the negative CT signals were observed, which was conducive to sensitively detecting the tumor. To verify whether the produced gas was H_2_, the tumor tissue was removed and tested by a gas chromatography system. As shown in Figure 3E, the spectrum displayed the peak of H_2_ at 1.2 min and the enlarged image further verified the existence of H_2_ (Figure 3F). Finally, the specific CT values in the region of interest (red circle) were then measured. It was observed that the CT values initially increased and then decreased after the injection of Hf‐MOF@AB@PVP nanoparticles. The enhanced CT value in the region of interest was 364.4 HU and then decreased to 22.4 HU, resulting in a drop ratio of 93.9% (Figure 3G). Furthermore, the decreasing trend was simulated to be a Boltzmann model. In comparison to the aqueous solution, the different downward trend may be attributed to the pressure inside the tumor. The contrast‐to‐noise ratio (CNR) was further calculated. Herein, CNR was calculated using the following equation: CNR = (CT Value_ROI_ – CT Value_muscle_)/SD_air._ As shown in Figure S13 (Supporting Information), the value of CNR changed from 0.49 (Pre) to 56.01 (0 min) to −29.15 (120 min), making it more conducive to diagnosing tumor. In summary, Hf‐MOF@AB@PVP nanoparticles had the capability to enhance both positive and negative CT imaging contrast, enabling accurate and specific tumor diagnosis.

### 2.4 CT Imaging Orthotopic Hepatocellular Carcinoma

**Figure 4:**
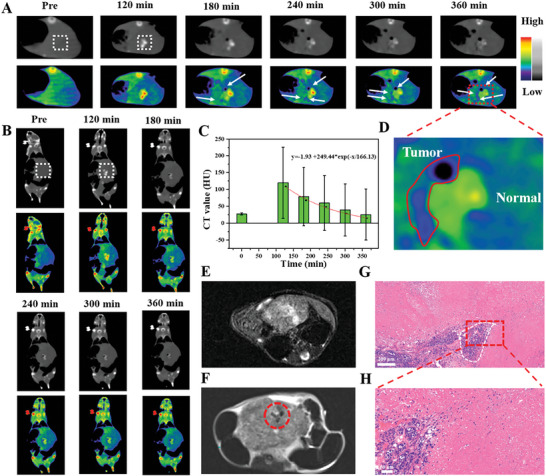
Transverse section CT images (grayscale in top and pseudo‐color in bottom) of mice (*n* = 3) intravenously injected with Hf‐MOF@AB@PVP nanoparticles in an orthotopic tumor model and in different time (A) and the white rectangular regions were the regions of interest and the regions of arrows were negative contrast areas; Coronal section CT images of mice intravenously injected with Hf‐MOF@AB@PVP nanoparticles in an orthotopic tumor model and in different time (B) and the white rectangular regions were the regions of interest; Histograms of CT value in the regions of interest and different time after the injection of Hf‐MOF@AB@PVP nanoparticles (C); An amplified CT image of the tumor at 360 min (D), and the negative contrast areas were tumor tissues and the positive contrast areas were normal liver tissues; MRI images of the mice at pre (E) and 360 min (F) and the red circle area was H_2_; H&E staining of hepatocellular carcinoma tissues (G) and an enlarged area (H), and the scale bar was 200 and 50 µm, respectively.

Hf‐MOF@AB@PVP nanoparticles were utilized for CT imaging of orthotopic hepatocellular carcinoma. The nanoparticles were intravenously injected into the mice for CT imaging. From the transverse CT imaging photos (grayscale and pseudo‐color) of mice (**Figure** **4**A), it was found that the CT value in the liver increased at 120 min following the injection. The corresponding CT value in the region of interest ranged from 27.4 to 120.3 HU. However, it could not distinguish the normal liver and tumor tissues. Over time, negative CT contrast signals (white arrow areas) began to appear and became more prominent around the positive CT contrast signals. It was because the nanoparticles encountered the acidic environment of hepatocellular carcinoma and released H_2_, which generated the negative CT imaging contrast. Furthermore, the coronal section CT images also demonstrated that Hf‐MOF@AB@PVP nanoparticles were concentrated in the liver (Figure 4B). The nanoparticles could create positive and negative CT contrast in normal liver tissues and tumor tissues respectively, achieving a sensitive detection of the liver tumor. After 360 min, the CT value in the region of interest turned out to be 25.4 HU with a decreased ratio was 78.9%, which was suitable for an exponential decay model (Figure 4C). The CT value in the negative area was −96.0 HU, which decreased by 450.3% in contrast to that in pre. The difference between negative and positive CT signals was high 216.3 HU, creating a significant contrast. From the pseudo‐color images, the contrast between positive and negative was more significant. The corresponding CNR value varied from 0.90 (Pre) to 23.17 (120 min) to −29.73 (360 min) (Figure S14, Supporting Information). More importantly, as shown in the enlarged pseudo‐color image at 360 min, most negative CT signals appeared on the left side of positive CT signals (Figure 4D). Whereas few negative CT signals were presented on the right side of positive CT signals. This observation suggested that the tumor was situated on the left side, while the right side corresponded to normal tissue in this study.

Since the magnetic resonance imaging (MRI) technique is sensitive to soft tissue imaging, it was utilized to verify the position of H_2_. As shown in Figure 4E (pre), little gas was observed within the tumor. After Hf‐MOF@AB@PVP nanoparticles were in the liver for 360 min, gas was generated in the tumor (Figure 4F). This was further confirmed that Hf‐MOF@AB@PVP nanoparticles generated H_2_ after responding to the acidic microenvironment in the tumor. Subsequently, the liver tissue was stained with hematoxylin‐eosin (H&E). The image showed the presence of hepatocellular carcinoma, facilitating its differentiation from normal liver tissues (Figure 4G). An enlarged image and a detailed drawing of the tumor validated the presence of hepatocellular carcinoma (Figure 4H).

### 2.5 Biocompatibility Evaluation of Hf‐MOF@AB@PVP Nanoparticles In Vivo

At last, the biocompatibility of Hf‐MOF@AB@PVP nanoparticles to institute of cancer research (ICR) mice was investigated. First, the mice were divided into 3 groups including the control group injected with normal saline, and the experiment groups injected with nanoparticles at 3 days and 30 days. Body weight variations were recorded over one month (Figure S15, Supporting Information). The weight growth trend in the control group and experimental group (30 days) remained consistent. The main organ tissues including the heart, liver, spleen, lung, and kidney were isolated and stained by H&E (Figure S16, Supporting Information). The results demonstrated little difference between the control and experimental groups. Furthermore, the blood parameters and serum biochemical analysis exhibited negligible side effects of the nanoparticles (Figure S17, Supporting Information). At last, the metabolism and biodistribution of Hf‐MOF@AB@PVP nanoparticles were investigated. As shown in Figure S18 (Supporting Information), it indicated that Hf‐MOF@AB@PVP nanoparticles were mainly metabolized through feces (relative changes based on the content of Hf in urine on the first day). Subsequently, the amount in several main organs indicated the nanoparticles mainly accumulated in the liver and spleen (Figure S19, Supporting Information). All of the above results indicated the acceptable biocompatibility of Hf‐MOF@AB@PVP nanoparticles as CT imaging contrast agents.

## 3 Conclusions and Discussions

In conclusion, a nanoagent fusing positive and negative CT contrast was presented to sensitively detect hepatoma. The nanoagent Hf‐MOF@AB@PVP was synthesized, which could accumulate in the liver and showed a positive CT contrast signal of 120.3 HU. Subsequently, the nanoagent exhibited a specific response to the acidic microenvironment in the tumor, generating H_2_ and causing a significant reduction in the CT value from 27.4 HU to −96.0 HU, representing a decrease of 450.3%. Therefore, the signal difference between the negative contrast area and the positive contrast area was 216.3 HU, forming a distinct contrast. More importantly, the relative position between the negative and positive contrast area could sensitively determine that the hepatoma tissues located on the left side of the normal liver tissues in this work, which was helpful to sensitively distinguish tumor from normal liver tissue utilizing CT imaging.

CT imaging possesses inherent spatial and temporal resolution capabilities. However, it is hampered by its limited sensitivity to soft tissue, a significant drawback.^[^ ^8^ ^]^ The strategy in this work was of great significance for accurately CT diagnosing soft tissue lesions by generating a distinct difference between positive and negative contrast in situ. The combination of positive and negative signals was like the “lunar eclipse”, and the around other positive signals were like “stars”. Therefore, the “lunar eclipse” was more obvious. Moreover, this work further expanded the scope of negative CT contrast agents in detecting solid tumors without cavities.

Although this nanoagent could be used to detect orthotopic hepatocellular carcinoma sensitively, there were still some considerations in this work. Gas known for its easy diffusion may cause interference in the detection of hepatocellular carcinoma. However, it was found that H_2_ accumulated around the nanoparticles and showed minimal diffusion in this study, which may be attributed to the generation rate of H_2_ and the pressure within the tumor. In both the subcutaneous xenograft and orthotopic tumor models, the declining trend of CT value was inconsistent, possibly due to variations in tumor pressure. Expanding on this point, if the pressure difference between tumors and normal tissues leads to varying rates of gas diffusion, it might be capable of detecting tumor boundaries based on their different diffusion rates of gas. In Figures 3 and 4, H_2_ was generated dynamically over time, resulting in a reduction of CT value in the tumor. The dynamic reduction of CT value had a close relationship with the acidic tumor environment. Alternatively, the combination of positive and negative CT contrast agents also brought some opportunities to develop ratiometric CT probes for detecting the microenvironment, such as hydrogen peroxide or carbonate ions, by releasing oxygen or carbon dioxide.

A typical single CT contrast agent provided one dimension of signal magnitude, which could be regarded as a scalar. In this study, the dual‐contrast nanoagent could provide both signal magnitude and direction information, similar to a vector. Therefore, the positive and negative CT contrast nanoagents can be, to some extent, considered as a vector CT contrast nanoagent. Actually, CT contrast nanoagents are usually heterogeneously distributed in solid tumors. A single CT contrast nanoagent can only show the tissues where the nanoagent was located, making it challenging to visualize the benign and malignant tissues surrounding the nanoagent. In this study, the relative position between the negative and positive contrast area was helpful in sensitively distinguishing hepatoma tissues from normal liver tissue (Figure 4).

In addition, due to the limited soft‐tissue resolution of CT and the uneven distribution of nanoagent in the liver, observing the precise position of hepatoma still faces certain limitations utilizing CT imaging. This issue might be addressed in the future through the utilization of spectral CT technology or photon CT technology.^[^ ^9^ ^]^ Furthermore, the heterogeneous distribution of the CT contrast nanoagent in the liver might bring contingency to the results in this study. Therefore, further optimization of the nanoagent is necessary to achieve a more uniform distribution.

## 4 Experimental Section

### Materials

Hafnium (IV) chloride, 2‐amino terephthalic acid (2‐ATPA), benzoic acid, *N*, *N*‐dimethylformamide (DMF), borane‐ammonia (AB) complex, and polyvinylpyrrolidone (PVP) (M.W. 10000) were purchased from Tansoole.

### Characterization

Transmission electron microscope (TEM) images were conducted on a TEM (JEOL LEM‐2100) at 200 kV (JEOL Ltd., Japan). Scanning electron microscope (SEM) images were carried on an SEM (Ultra 55, Zeiss). X‐ray diffraction (XRD) results were obtained on a RigakuUltima IV X‐ray diffractometer instrument (Cu Kα (λ = 1.5405 Å) along with a scan range from 10° to 60°. Hydrodynamic size and Zeta potentials were conducted on an analyzer (Malvern instrument ZS90). Fourier transform infrared (FT‐IR) spectra and in situ FT‐IR spectra with varied temperature were acquired on a spectrophotometer (Nicolet 6700, Thermofisher) utilizing KBr. Raman spectra were conducted on a Raman spectrometer (XploRA, HORIBA JobinYvon). X‐ray photoelectron spectroscopy (XPS) was conducted on a machine (Thermo Scientific K‐Alpha, America). Nitrogen adsorption and desorption experiments were carried out on a BET surface area and porosity analyzer (ASAP2020). Thermogravimetry (TG) was conducted on an analyzer (STA449F3). Gas analysis was operated on a gas chromatography system (GC2060). CT experiments were obtained on a CT machine (SOMATOM Force, SIEMENS).

### Preparation of Hf‐Based Metal–Organic Frameworks (Hf‐MOF)

Hf‐MOF nanoparticles were prepared according to a synthesis method of Zr‐based metal–organic frameworks with some modifications. First, HfCl_4_ (0.343 mmol) and 3.43 mmol of benzoic acid were dispersed in 20 mL of DMF in a 100 mL Teflon‐based flask. 0.343 mmol of the linker (2‐aminoterephthalic acid) was then added into the above system with ultrasound for 3 min. 0.025 mL of purified water was added into the solution. The system was transferred and kept in an oven at 120 °C for 24 h. Twenty four hours later, the solution was cooled to room temperature and the precipitates were isolated by centrifugation. The precipitates were washed 2 times by DMF and water, respectively. The resulting Hf‐MOF nanoparticles were dispersed in 10 mL of water.

### Preparation of Hf‐MOF@AB@PVP

One hundred milligrams of AB molecules were added into the above Hf‐MOF solution using ultrasound. This system was then stirred at room temperature for 24 h. After 24 h, 200 mg of PVP was added into the solution and continued to be stirred for another 24 h. Finally, the precipitates were isolated by centrifugation and washed with water. The resulting Hf‐MOF@AB@PVP nanoparticles were freeze‐dried to be powders under a vacuum.

### Investigation of Hf‐MOF@AB@PVP Nanoparticles with Different pH Values

1.0 mg mL^−1^ of Hf‐MOF@AB@PVP nanoparticles were redispersed in a solution (50 mL) with different pH values (pH = 5.5, 6.5, or 7.4) at different time points. First, the standard curve of H_2_ release was drawn by quantifying the volume of H_2_ using gas chromatography. The released H_2_ was collected by a microinjector and injected into the gas chromatography. Finally, the amount of H_2_ released was calculated by the ideal gas equation (pV = nRT), wherein, p is pressure, V is the volume of gas, n is the amount of gas, R is the molar gas constant and T is temperature.

### CT Imaging of Hf‐MOF@AB@PVP Nanoparticles in Aqueous Solution

First, different concentrations of Hf‐MOF@AB@PVP nanoparticles (2000, 1000, 5000, 250, and 125 ppm) were CT imaged by a dual‐energy CT imaging machine (100 mAs, 70/Sn 150 kV). Subsequently, the CT imaging capability of Hf‐MOF@AB@PVP nanoparticles (500 ppm) with different pH values (pH = 5.5, 6.5, or 7.4) at different time points was investigated. The CT imaging conditions were identical.

### CT Imaging of Hf‐MOF@AB@PVP Nanoparticles In Vivo

All animal experiments were conducted according to the guidelines of the Regional Animal Experiment Ethics Committee and the nursing regulations were authorized by the Experimental Animal Management Committee of Tongji University Affiliated Shanghai 10th People's Hospital (certification number: SHDSYY‐2021‐T0043). First, 3 subcutaneous tumor‐bearing mice were constructed. 10^6^ of Huh 7 cells were injected into each Balb/c nude mouse (6 weeks, male) subcutaneously. Ten days later, the models were constructed successfully. Then, 100 µL of Hf‐MOF@AB@PVP nanoparticles (10000 ppm) were injected into the muscle tissues of mice (3) and the tumor tissues of mice (3), respectively, to investigate the H_2_ release and CT imaging at different time points. To ensure comparability, the CT imaging of mice should be at the same level as much as possible.

Subsequently, 10^6^ of Huh 7 cells were injected into the liver of each Balb/c nude mouse (6 weeks, male, in total 3) in situ. Ten days later, the models were constructed. Then, 100 µL of Hf‐MOF@AB@PVP nanoparticles (25 000 ppm) were intravenously injected into each mouse (100 mg kg^−1^). CT imaging was conducted at different time points to investigate the nanoparticles’ imaging capability.

### Biocompatibility Evaluation of Hf‐MOF@AB@PVP Nanoparticles In Vivo

Fifteen normal Institute of Cancer Research (ICR) mice (6 weeks, male) were purchased and raised for one week to adapt to the new environment. One week later, the mice were divided into 3 groups, including the control group (*n* = 5), the group (*n* = 5) of injected nanoparticles for 3 days, and the group (*n* = 5) of injected nanoparticles for 30 days. 100 µL of Hf‐MOF@AB@PVP nanoparticles (25 000 ppm) were intravenously injected into the experiment group (100 mg kg^−1^). The weight of the mice in each group was then recorded for 30 days. The mice's blood was extracted from the mice's eyes. Subsequently, the main organs including the heart, liver, spleen, lung, and kidney were dissected to be stained by hematoxylin‐eosin (H&E) for histological analysis.

Subsequently, 9 healthy BALB/c mice were divided into 3 groups. Hf‐MOF@AB@PVP nanoparticles (100 mg kg^−1^, Hf) were intravenously into each mouse. The urine and feces were collected at different time points (24, 72, and 240 h), respectively. The mice were then sacrificed to collect their main organs (heart, liver, spleen, lung, and kidney), which were next dissolved by aqua regia.

### Statistical Analysis

For normally distributed data, a two‐tailed t‐test was used. For non‐normally distributed data, the Mann‐Whitney‐u test was used. *p* < 0.05 was considered statistically significant.

## Conflict of Interest

The authors declare no conflict of interest.

## Supporting information

Supporting Information

## Data Availability

The data that support the findings of this study are available from the corresponding author upon reasonable request.
