# Supplementary material for: Fusing Positive and Negative CT Contrast Nanoagent for the Sensitive Detection of Hepatoma
Source: Adv Sci (Weinh). 2023 Oct 23;10(34):2304668. doi: 10.1002/advs.202304668 (PMC10700169; doi:10.1002/advs.202304668)
Supplement: Supplementary file 1 — Supporting Information [file ADVS-10-2304668-s001.pdf]

ADVANCED SCIENCE
Open Access

Supporting Information

for *Adv. Sci.*, DOI 10.1002/advs.202304668

Fusing Positive and Negative CT Contrast Nanoagent for the Sensitive Detection of Hepatoma

Xianfu Meng, Jiahao Gao, Yanhong Sun, Fei Duan, Bixue Chen, Guanglei Lv, Huiyan Li, Xingwu Jiang, Yelin Wu, Jiawen Zhang, Xiangming Fang*, Zhenwei Yao, Changjing Zuo* and Wenbo Bu**

Supporting Information

Fusing Positive and Negative CT Contrast Nanoagent for the Sensitive Detection of Hepatoma

Xianfu Meng, Jiahao Gao, Yanhong Sun, Fei Duan, Bixue Chen, Guanglei Lv, Huiyan Li, Xingwu Jiang, Yelin Wu, Jiawen Zhang, Xiangming Fang,* Zhenwei Yao, Changjing Zuo,* Wenbo Bu**

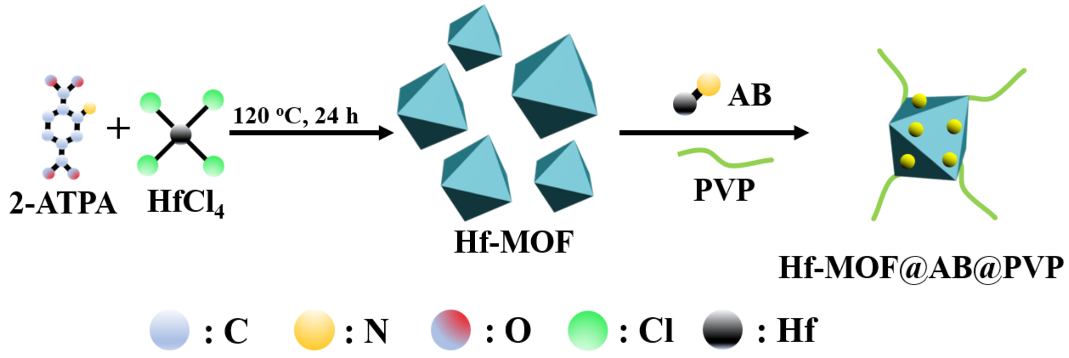

Figure S1. A synthesis method of Hf-MOF nanoparticles.

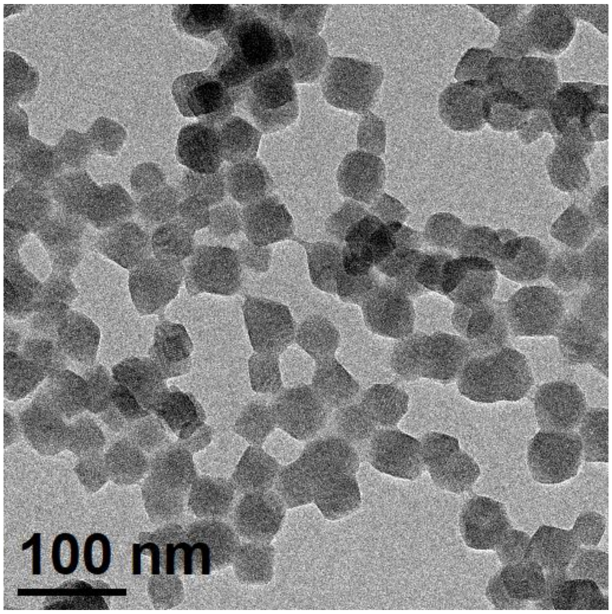

Figure S2. TEM image of Hf-MOF nanoparticles.

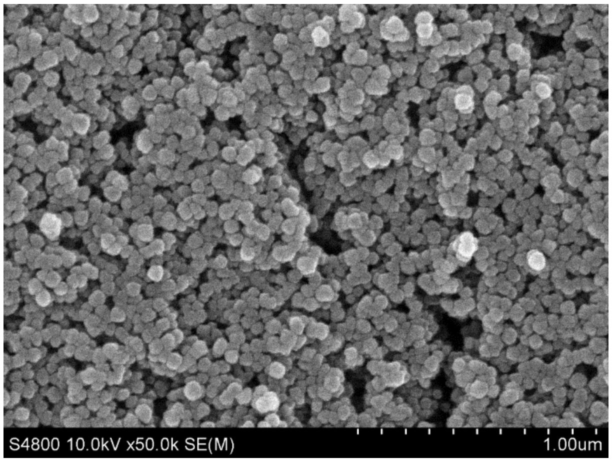

Figure S3. SEM image of Hf-MOF nanoparticles.

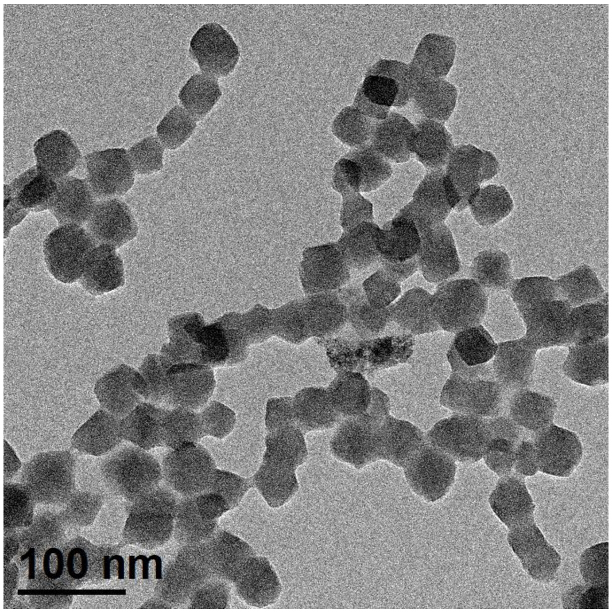

Figure S4. TEM image of Hf-MOF@AB nanoparticles.

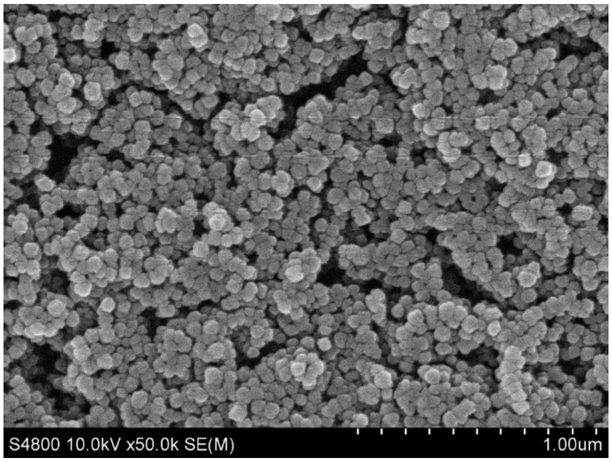

Figure S5. SEM image of Hf-MOF@AB nanoparticles.

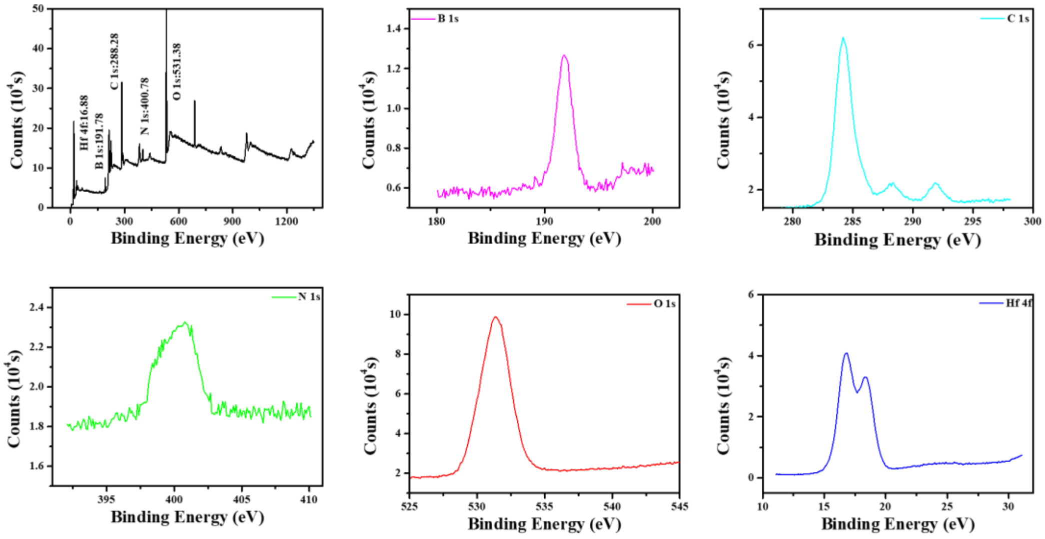

Figure S6. X-ray photoelectron spectroscopy (XPS) spectrum of Hf-MOF@AB@PVP and the XPS spectra of B, C, N, O and Hf elements.

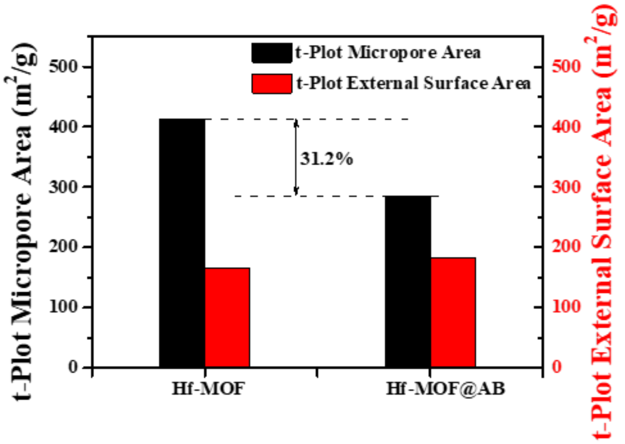

Figure S7. T-plot micropore area and t-plot external surface area of Hf-MOF and Hf-MOF@AB.

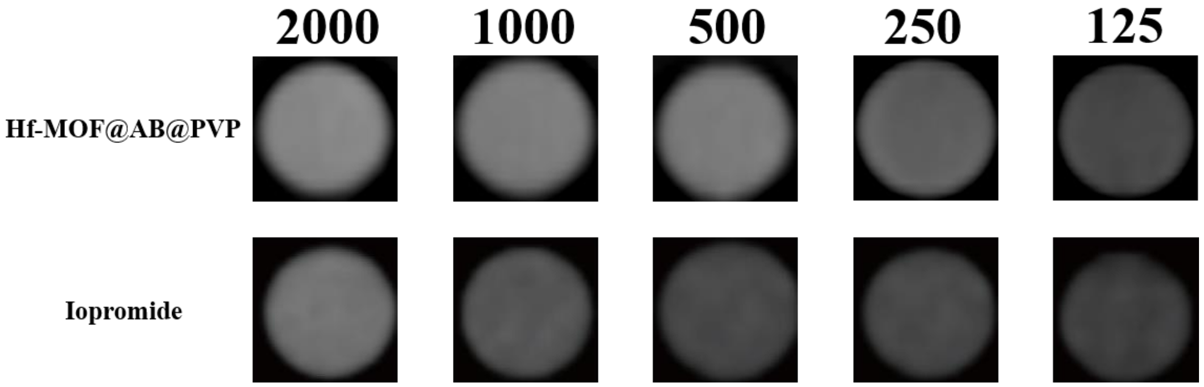

Figure S8. CT imaging capability of Hf-MOF@AB@PVP nanoparticles and clinical iodine (iopromide) at different concentrations (2000, 1000, 500, 250, and 125 ppm).

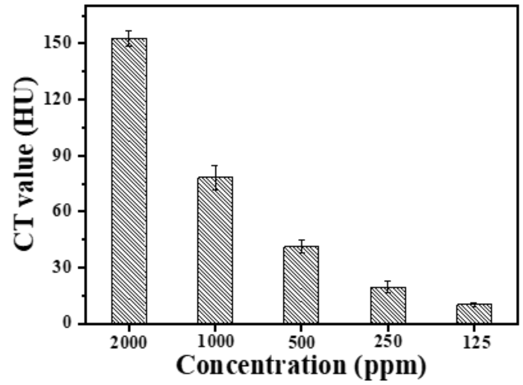

Figure S9. Histograms of corresponding CT value of Hf-MOF@AB@PVP nanoparticles at different concentrations.

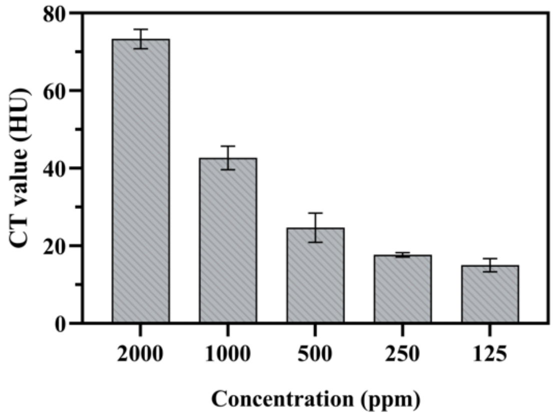

Figure S10. Histograms of corresponding CT value of clinical iodine (iopromide) at different concentrations.

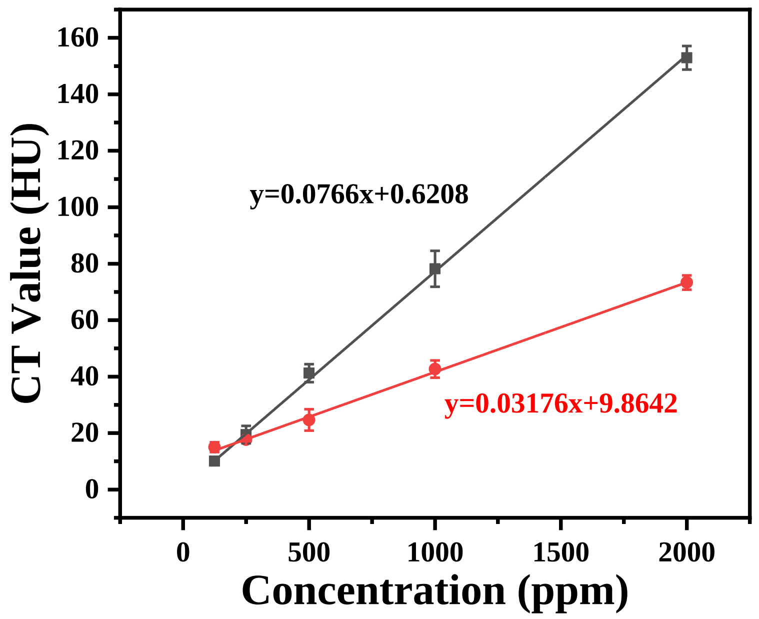

Figure S11. CT value dot plot of Hf-MOF@AB@PVP nanoparticles and clinical iodine (iopromide) at different concentrations (2000, 1000, 500, 250, and 125 ppm).

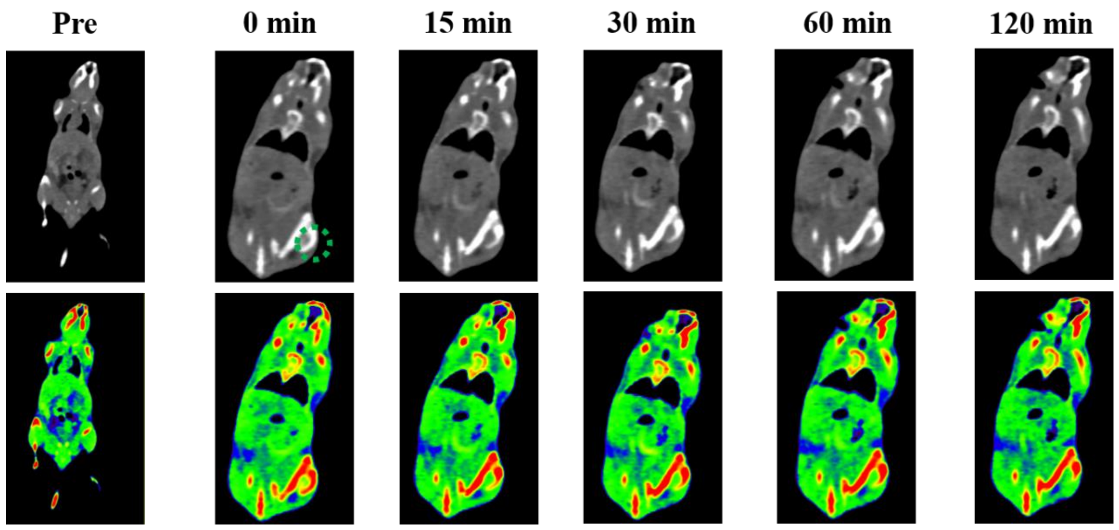

Figure S12. Coronal section CT images of mice after Hf-MOF@AB@PVP nanoparticles were injected into the muscles and the green circle was the region of interest.

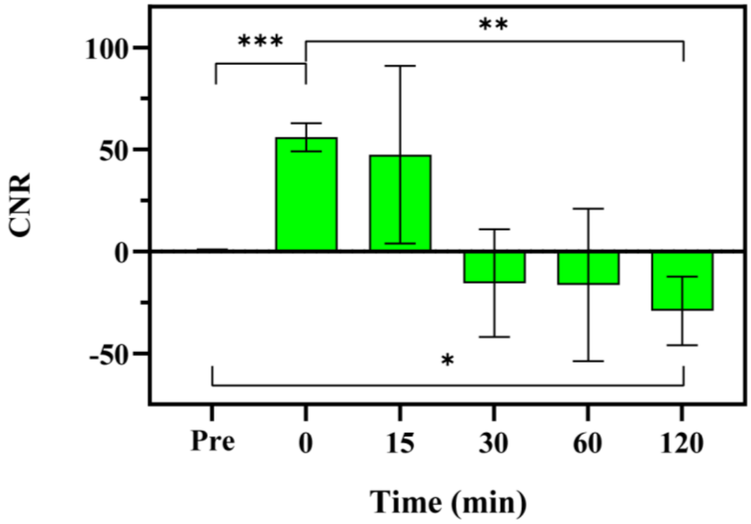

Figure S13. CNR value of the tumor after being intratumorally injected with Hf-MOF@AB@PVP nanoparticles. For normally distributed data, a two-tailed t-test was used. For non-normally distributed data the Mann-Whitney-u test was used. $p<0.05$ was considered statistically significant. * for $p<0.05$, ** for $p<0.01$, *** for $p<0.001$.

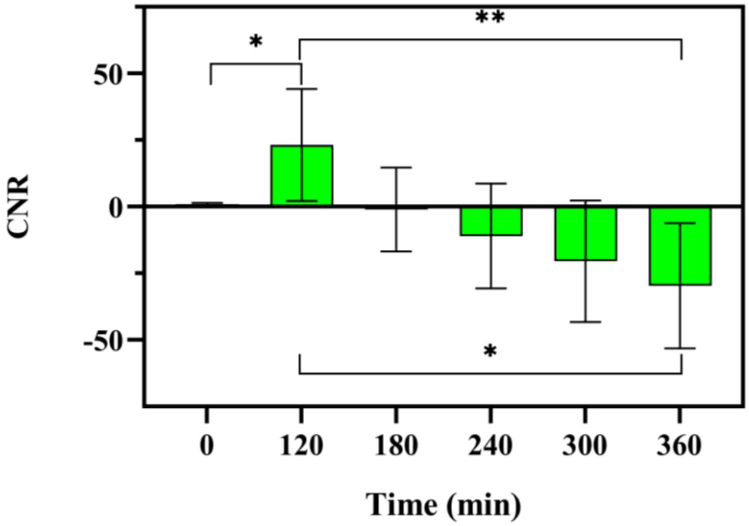

Figure S14. CNR value of HCC in situ after being intravenously injected with Hf-MOF@AB@PVP nanoparticles.

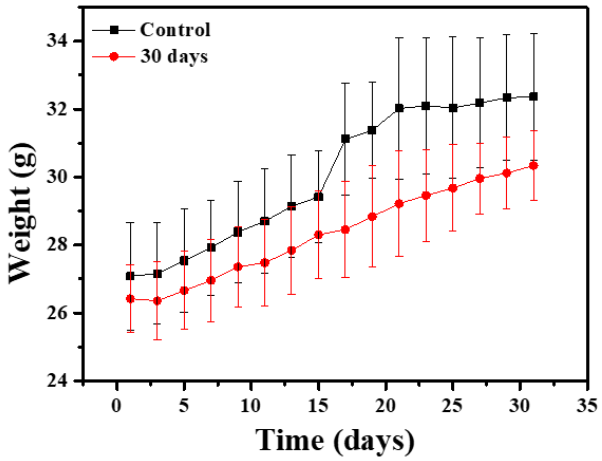

Figure S15. Body weight variations of the control and experiment groups.

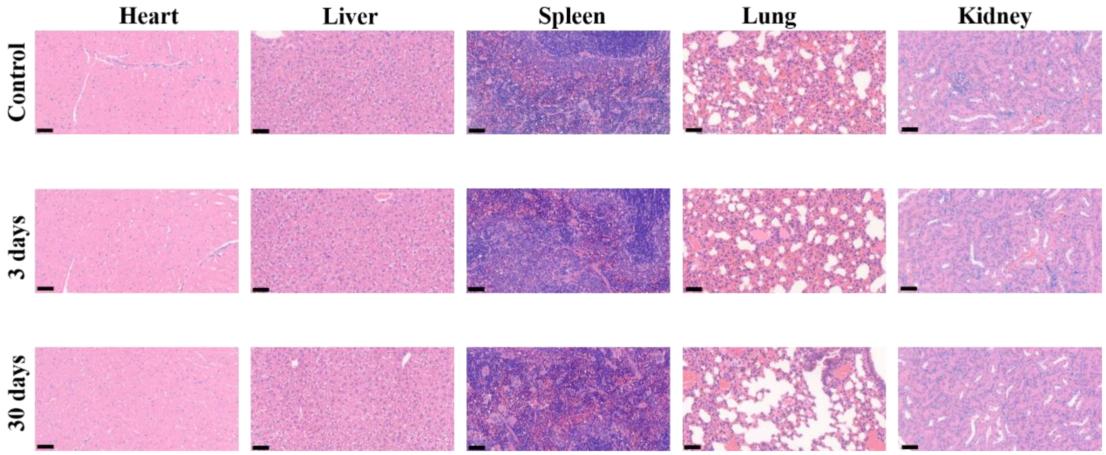

Figure S16. H&E staining of main organs including heart, liver, spleen, lung and kidney in the control and experiment groups. The scale bar was 50 μm.

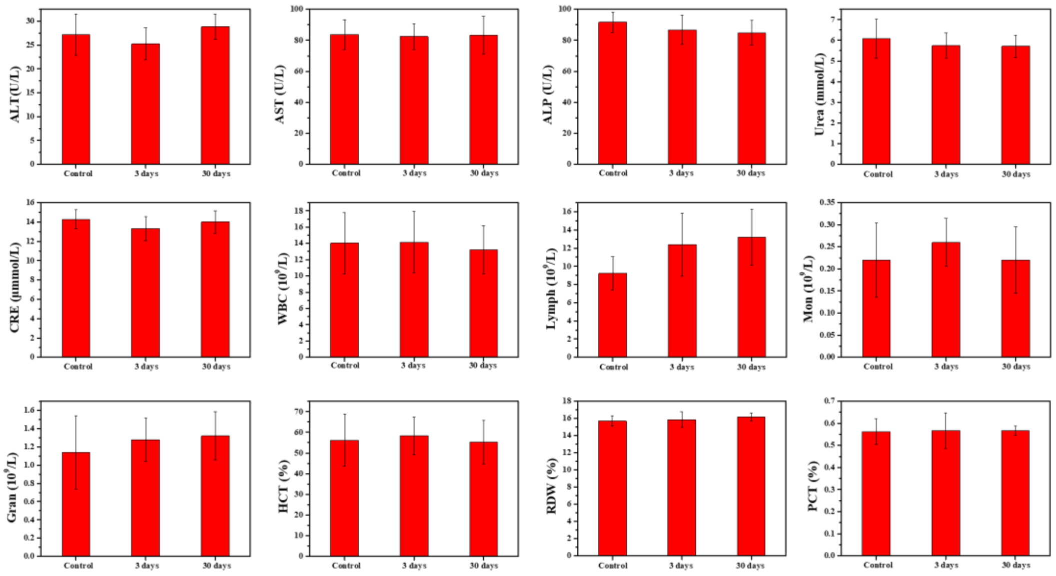

Figure S17. Serum biochemistry and hematology analysis in control and the experiment groups (3 and 30 days).

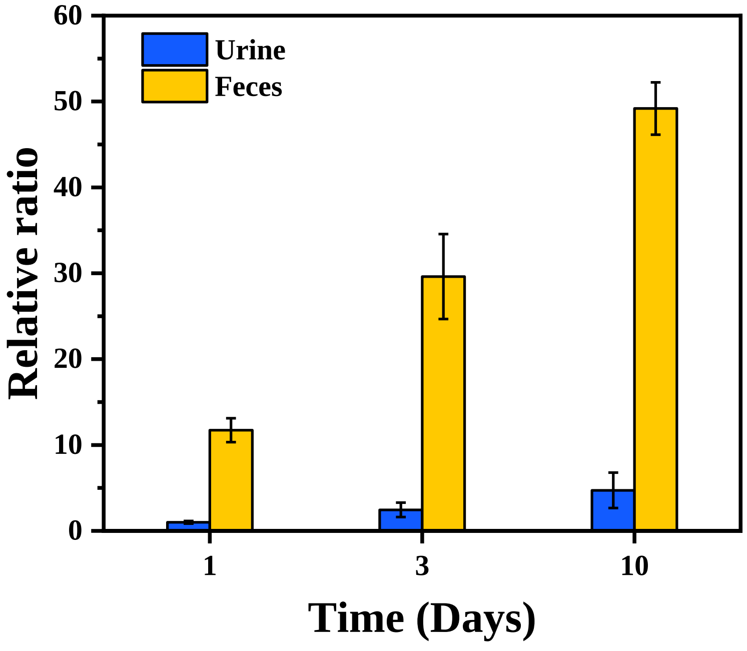

Figure S18. The metabolism kinetics of Hf-MOF@AB@PVP in feces and urine at 24 h, 72 h, and 240 h time points.

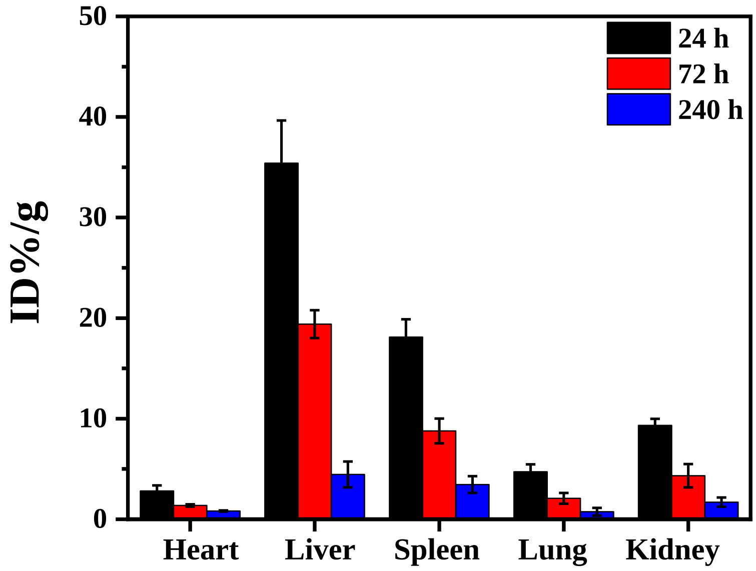

Figure S19. The in vivo biological distribution of Hf-MOF@AB@PVP nanoparticles at 24 h, 72 h, and 240 h time points.
